# Supplementary figures and images for: Wastewater-integrated pathogen surveillance dashboards enable real-time, transparent, and interpretable public health risk assessment and dissemination
Source: PLOS Glob Public Health. 2025 May 5;5(5):e0004443. doi: 10.1371/journal.pgph.0004443 (PMC12052137; doi:10.1371/journal.pgph.0004443)

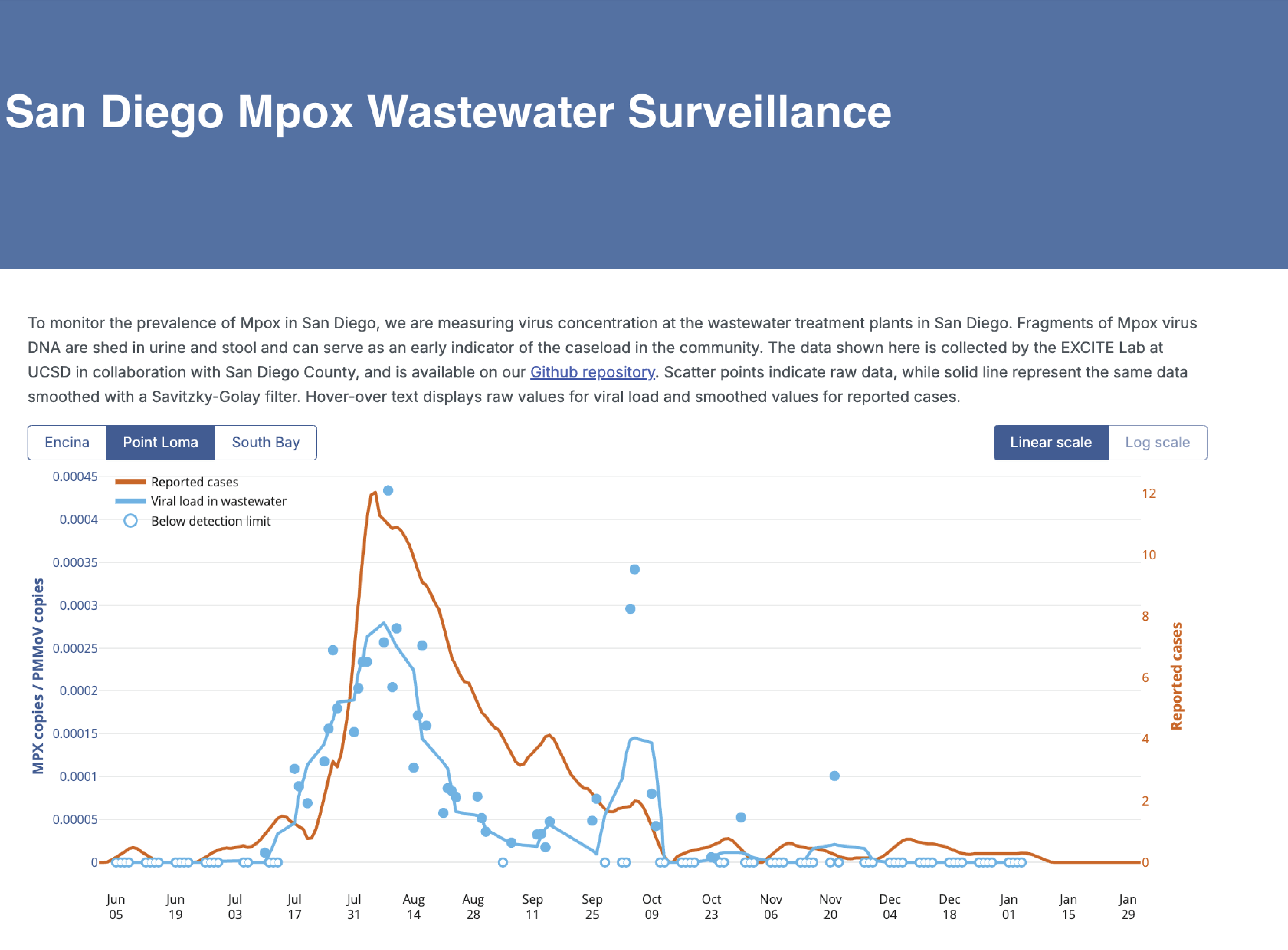

Supplement: S1 Fig — This code is modular and resulting plots can be easily inserted as an iframe. (PNG) [file pgph.0004443.s001.png]

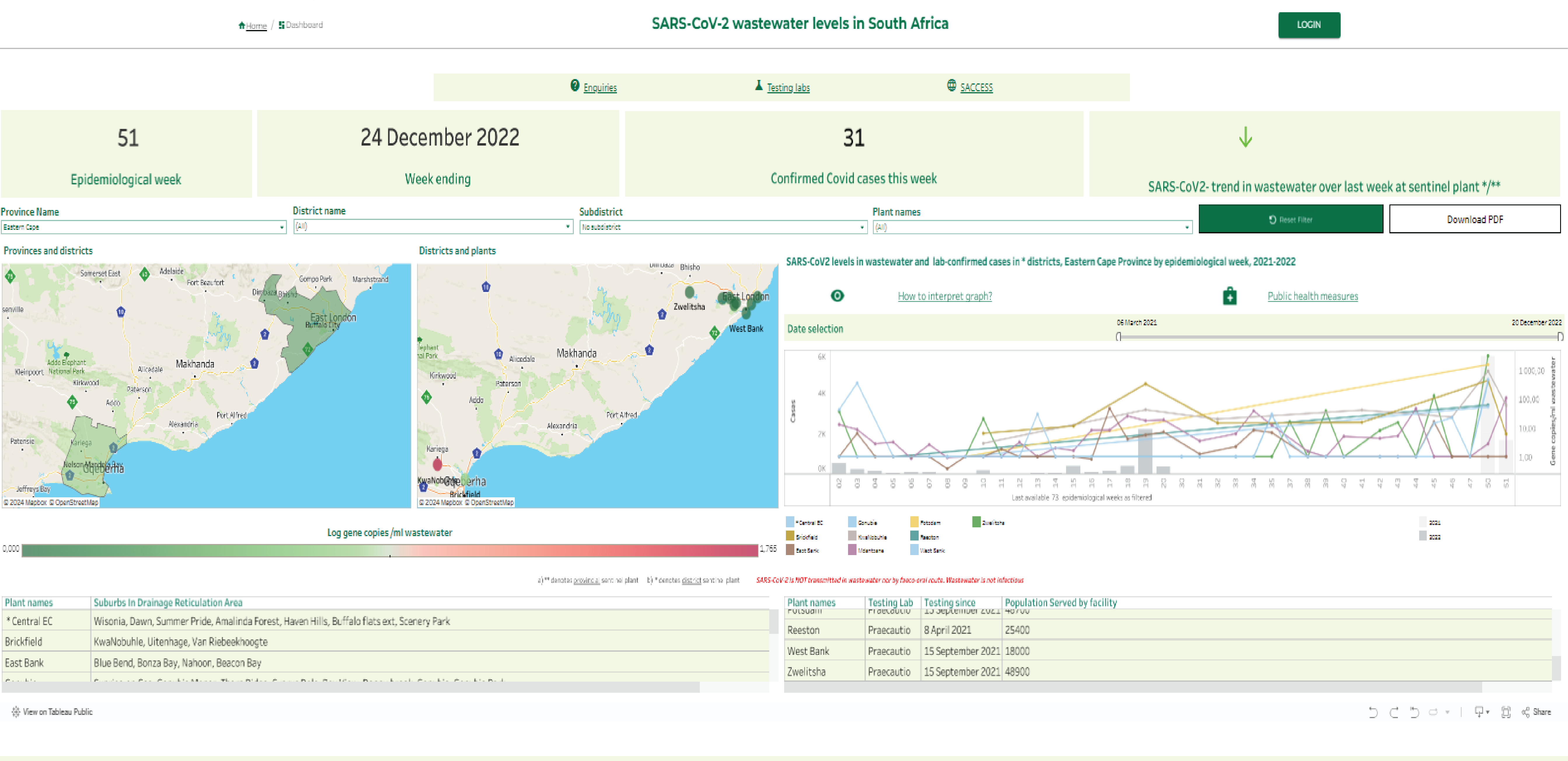

Supplement: S2 Fig — Key metrics providing the total number of confirmed clinical cases reported together with any increase or decrease in viral loads detected from wastewater. The dashboard also provided provincial and district level maps and viral load analyses from various WWTPs. Maps used are open data from OpenStreetMap(openstreetmap.org) available under the Open Data Commons Open Database License (https://opendatacommons.org/licenses/odbl/). (PNG) [file pgph.0004443.s002.png]

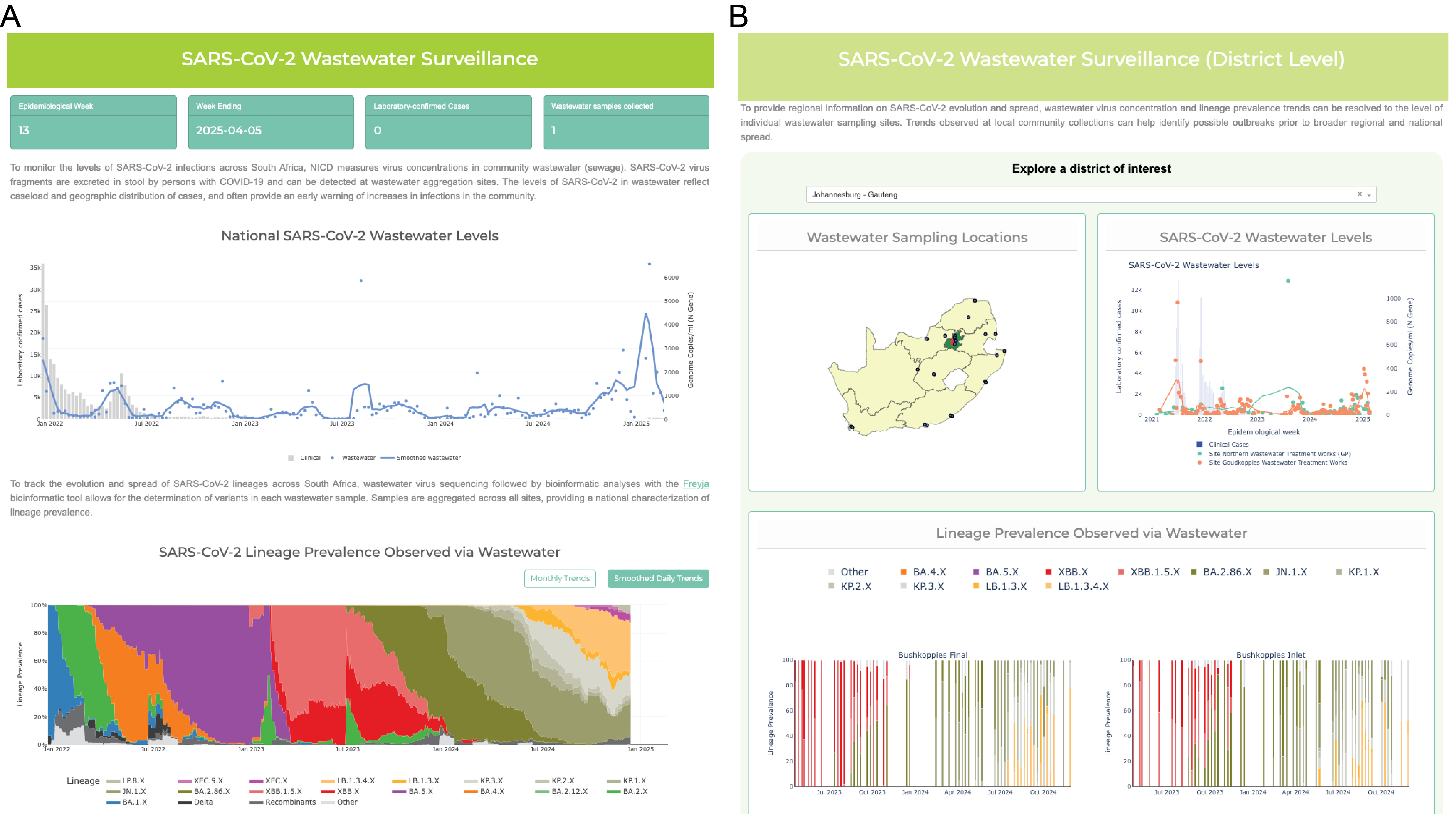

Supplement: S3 Fig — A. Main page containing surveillance data from across the country, along with a description of wastewater surveillance efforts and their significance. B. District level dashboard includes a brief description of the available resolution provided by the program as well as a pull-down selector to access data for each monitored district. Province shapefiles were obtained under an open CC-BY 4.0 license [26], https://simplemaps.com/static/svg/country/za/admin1/za.json. (PNG) [file pgph.0004443.s003.png]
